# *Echinochloa crus-galli* genome analysis provides insight into its adaptation and invasiveness as a weed

**DOI:** 10.1038/s41467-017-01067-5

**Published:** 2017-10-18

**Authors:** Longbiao Guo, Jie Qiu, Chuyu Ye, Gulei Jin, Lingfeng Mao, Haiqiang Zhang, Xuefang Yang, Qiong Peng, Yingying Wang, Lei Jia, Zhangxiang Lin, Gengmi Li, Fei Fu, Chen Liu, Li Chen, Enhui Shen, Weidi Wang, Qinjie Chu, Dongya Wu, Sanling Wu, Chenyang Xia, Yongfei Zhang, Xiaomao Zhou, Lifeng Wang, Lamei Wu, Weijie Song, Yunfei Wang, Qingyao Shu, Daisuke Aoki, Emi Yumoto, Takao Yokota, Koji Miyamoto, Kazunori Okada, Do-Soon Kim, Daguang Cai, Chulong Zhang, Yonggen Lou, Qian Qian, Hirofumi Yamaguchi, Hisakazu Yamane, Chui-Hua Kong, Michael P. Timko, Lianyang Bai, Longjiang Fan

**Affiliations:** 1 China National Rice Research Institute, Chinese Academy of Agricultural Sciences, Hangzhou, 310006 China; 2 Institute of Crop Science and Institute of Bioinformatics, Zhejiang University, Hangzhou, 310058 China; 3 Guhe Information, Hangzhou, 310058 China; 4 College of Resources and Environmental Sciences, China Agricultural University, Beijing, 100193 China; 5 Hunan Weed Science Key Laboratory, Hunan Academy of Agriculture Science, Changsha, 410125 China; 6 Analysis Center of Agrobiology and Environmental Sciences, Faculty of Agriculture, Life and Environmental Sciences, , Zhejiang University, Hangzhou, 310058 China; 7 State Key Laboratory of Rice Biology, Institute of Biotechnology, Zhejiang University, Hangzhou, 310058 China; 8 Zhejiang Sheng Ting Biotechnology Co., Ltd., Taizhou, 318020 China; 9 Department of Bioscience, Faculty of Biotechnology, Fukui Prefectural University, Fukui, 910-1195 Japan; 10 Department of Biosciences, Teikyo University, Tochigi, 320-8551 Japan; 11 Biotechnology Research Center, The University of Tokyo, Tokyo, 113-8657 Japan; 12 Department of Plant Science, Institute of Agriculture and Life Sciences, College of Agriculture and Life Sciences, Seoul National University, Seoul, 151-921 Korea; 13 Department of Molecular Phytopathology and Biotechnology, Christian-Albrechts-University of Kiel, Kiel, D-24118 Germany; 14 Faculty of Agriculture, Tokyo University of Agriculture, Kanagawa, 243-0034 Japan; 15 Department of Biology, University of Virginia, Charlottesville, VA 22904 USA

## Abstract

Barnyardgrass (*Echinochloa crus-galli*) is a pernicious weed in agricultural fields worldwide. The molecular mechanisms underlying its success in the absence of human intervention are presently unknown. Here we report a draft genome sequence of the hexaploid species *E. crus-galli*, i.e., a 1.27 Gb assembly representing 90.7% of the predicted genome size. An extremely large repertoire of genes encoding cytochrome P450 monooxygenases and glutathione S-transferases associated with detoxification are found. Two gene clusters involved in the biosynthesis of an allelochemical 2,4-dihydroxy-7-methoxy-1,4-benzoxazin-3-one (DIMBOA) and a phytoalexin momilactone A are found in the *E. crus-galli* genome, respectively. The allelochemical DIMBOA gene cluster is activated in response to co-cultivation with rice, while the phytoalexin momilactone A gene cluster specifically to infection by pathogenic *Pyricularia oryzae*. Our results provide a new understanding of the molecular mechanisms underlying the extreme adaptation of the weed.

## Abstract (web summary)

Barnyardgrass is a problematic agricultural weed. Here, via genomic analysis, Guo et al. identify genes potentially underlying its success, including a gene cluster activated in response to co-cultivation with rice that leads to synthesis of the allelochemical DIMBOA.

## Introduction

Agronomically important weeds cause tremendous worldwide losses of crop yields estimated at over $95 billion annually according to United Nations Food and Agriculture Organization^1^. Within the short period of agricultural history, weedy plants have exploited the human-mediated environment of farmers’ fields by undergoing rapid adaptive evolution that allowed them to proliferate and escape eradication using weediness traits such as rapid growth rates, prolonged dormancy, ease of dispersal, herbicide resistance, etc^2^. Thus, weeds have evolved to survive in agroecosystems under natural and unintentionally human-mediated selection^3^, leading to a distinct evolutionary state that has proceeded in parallel to crop domestication^2^. Therefore, agricultural weeds are ideal models for the study of environmental adaption of plants from an evolutionary perspective. Beyond purely academic interests, uncovering how weeds evolve is crucial for their management and protection of the global food supply.

In contrast to numerous food and forage crops where the genomes of many important domesticated forms and their wild relatives have been sequenced leading to a better understanding of the genetic basis of many important agronomic traits^4^, little genomic scale analysis has taken place on agricultural weed species despite their economic significance. In fact, among the important agricultural weeds known, only the dicot horseweed (*Conyza canadensis*) has had its whole genome sequenced thus far^5^.

The genus *Echinochloa* (Poaceae) or barnyardgrass includes approximately 250 annual and perennial species^6^. Systematic frequency assessments for rice weeds based on phytosociological studies showed that *Echinochloa* species are the most frequent weeds associated with rice cultivation globally^7^. Among *Echinochloa* species, *E. crus-galli* (annual, 2*n* = 6*x* = 54) is the most prevalent species^8, 9^, followed by *Echinochloa colona* and the tetraploid *Echinochloa oryzicola* ^7^. While conscious artificial selection for useful agronomical traits drove changes in early rice species during domestication, wild barnyardgrass growing in the same paddy fields was driven by natural and unintentional selections to evolve adaptive and competitive characteristics to evade removal from rice fields. For example, *E. crus-galli* in paddy fields morphologically resembles rice (*Oryza sativa*) at the seedling stage, which increase the difficulty of recognition during manual weeding^10^. More recently, *E. crus-galli* acquired or evolved target-site and non-target-site herbicide resistance making it among the most pernicious herbicide resistant weeds of the world^11^. Worldwide losses of yield in rice due to *E. crus-galli* competition are estimated to be about 35%^12^.

Allelopathy, or the ability of one plant to suppress the growth of another nearby plant through the release of chemical compounds (i.e., allelochemicals) in the rhizosphere is one of the most important weediness features. Allelopathy plays an important role in natural and agricultural ecosystems, particularly in crop ecosystems^13^ and has received much attention in recent years because it could potentially serve as an alternative weed management strategy in crop production without environmental cost^14^. Many field and greenhouse studies have demonstrated the allelopathic potential of rice cultivars on barnyardgrass^14^.

A number of plant secondary metabolites have been suggested to serve as allelochemicals and molecular genetic studies have shown that gene clusters are often responsible for the biosynthesis of the allelopathic compounds^15^. The first allelochemical gene cluster intensively studied was that involved in the formation of 2,4-dihydroxy-7-methoxy-1,4-benzoxazin-3-one (DIMBOA), a protective and allelopathic defense compound against weeds, present in maize (*Zea mays*)^16^. The maize DIMBOA gene cluster is located on Chromosome 4 and consists of eight genes (*BX1* to *BX8*)^16^. In rice, two gene clusters involved in the biosynthesis of the allelopathic diterpene phytoalexins momilactone A and phytocassane have been identified on Chromosomes 4 and 2, respectively^17, 18^. These diterpene phytoalexins can significantly inhibit growth of *E. crus-galli*.

Considerable attention has been devoted to understand allelopathic interactions between crops and weeds, but most studies have focused on the genetic mechanisms of the impact of crops on weeds. Knowledge of the underlying basis for how weeds respond to the allelopathic challenge from crops and why weeds are dominant in crop fields without human intervention is limited. In this study, we generated a draft genome assembly for the hexaploid species *E. crus-galli*, the dominant weed in rice paddy fields, and analyzed the dynamic changes in transcriptome profiles of *E. crus-galli* growing alone and in response to the co-cultivation with rice plants. In the genome, We have identified three copies of gene clusters involved in production of an allelochemical (DIMBOA) used by *E. crus-galli* against rice and one copy of a phytoalexin (momilactone A) gene cluster against blast disease (*P. oryzae*) in the paddy field environments.

## Results

### Genome assembly and annotation

The *E. crus-galli* line STB08, collected from rice paddy fields in the lower Yangtze River region of China, highly resembles cultivated rice in morphology (Supplementary Fig. 1), and has a chromosome number of 2*n* = 6*x* = 54. A total of 207.4 Gb of sequence data were generated using the Illumina HiSeq 2000 system from STB08 genomic DNA libraries with fragment sizes varying between 160 bp to 20 Kb (Table 1; Supplementary Table 1). In addition, the Pacbio RS II system was used to generate 32.9 Gb third-generation long reads, totally representing  ~ 171× coverage of the *E. crus-galli* genome estimated to be  ~ 1.4 Gb in size based on the *K*-mer analysis and flow cytometry (Supplementary Fig. 2). *De novo* assembly yielded a draft genome of 1.27 Gb, representing 90.7% of the *E. crus-galli* genome ( > 1 Kb), with a scaffold N50 length of 1.8 Mb. Five fosmid clones ( > 15 Kb) were sequenced and compared with the assembly, and is confirm to be of good consistence (Supplementary Table 2). About 92.3% of the core eukaryotic genes (CEGs) could be completely aligned with the *E. crus-galli* gene set. We have also used BUSCO to judge the assembly of *E. crus-galli*, and found that the ‘complete’ percent is 95.5%, which is comparable to that of *S. bicolor* (96.4%) and *S. italica* (94.3%) genome (Supplementary Table 3). In addition, we mapped 74 publicly available *E. crus-galli* EST sequences and 156,757 transcripts generated by this study to the assembled genome and observed high mapping and identity rates (Supplementary Table 3).

**Table 1:** Summary of the genome assembly and annotation of *E. crus-galli*

*Sequencing and assembly*				
Sequencing	Insert libraries	Illumina	Pacbio RS II	Total size (Gb)
	160 bp-20 Kb	148.2×	23.5×	240.3
Scaffold	N50 size (Mb)	N90 size (Kb)	The longest (Mb)	Total non-N size (Gb)
	1.80	453.89	11.70	1.27
*Genome annotation*				
Protein-coding gene	Gene models	Supported by homologs^a^	Supported by EST and Swiss-prot etc.^b^	Gene size (bp)
	108,771	74.2%	85.17%	1,901
Non-coding RNA	miRNA	tRNA	rRNA	snoRNA
	785	2306	1890	3378
Repetitive elements (%)	LTR	LINEs& SINEs	DNA transposon	Total
	21.9	2.5	8.0	40.7

^a^Annotated gene sets of six sequenced plants (*B. distachyon*, *O. sativa*, *S. bicolor*, *Z. mays*, *S. italic*, and *A. thaliana*)

^b^Swiss-prot, InterProt and nr databases are included

For gene annotation, transcriptomic data from the whole plant were generated by RNA-Seq (Supplementary Table 4). By integrating gene finding results from *ab initio*, homology- and transcript-based approaches, we predicted 108,771 protein-coding in the *E. crus-galli* genome (Table 1). Of the 108,771 genes, 85% were supported by either the identification of homologues in other species or RNA-Seq data. In addition to protein-coding genes, 785 microRNAs (miRNAs) and other non-coding RNAs were also identified in the *E. crus-galli* genome (Table 1 and Supplementary Table 5). On the basis of homology searches and *de novo* methods, we identified a total of 514 Mb of repetitive elements which represents 40.7% of the genomic assembly (Table 1). Among the repetitive sequences, long terminal retrotransposons (LTRs) were the most abundant, accounting for 21.9% of the assembly (Supplementary Table 6).

**Fig. 1:**
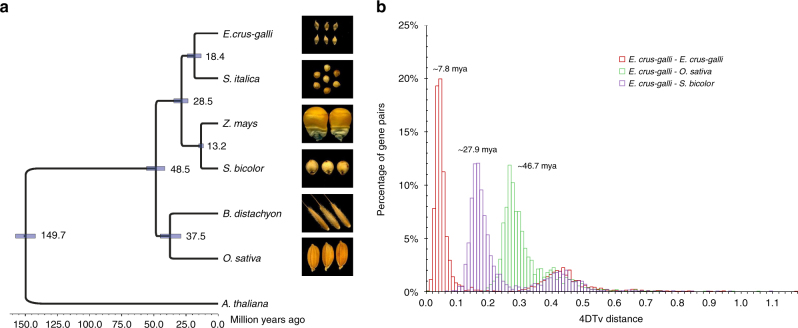
Phylogenetics of *E. crus-galli*. **a** Single-copy gene based phylogenetic tree and divergence times of *E. crus-galli* and other grasses with Arabidopsis as an outgroup. **b** Distribution of the transversion rates at the four-fold degenerate sites (4dTv) of paralogous gene pairs of *E. crus-galli* and other grasses. The times of *E. crus-galli* speciation and *E. crus-galli*-*Sorghum* and -*Oryza* divergence are given based on corresponding paralogous and orthologous peaks

Using pairwise protein sequence comparisons, putative orthologs and paralogs are analyzed among *E. crus-galli* and five other grass family members, including *B. distachyon*, *O. sativa*, *S. bicolor*, *Z. mays*, and *S. italica*. We found 26,679 gene families (containing a total of 72,363 genes) in the *E. crus-galli* genome, of which 6,789 families containing 20,739 genes appear to be *Echinochloa*-specific. On the basis of the orthologous single-copy genes, the *Oryza*- and *Sorghum*-*Echinochloa* divergence times were estimated to be ca. 48.5 and 28.5 million years ago (Mya), respectively (Fig. 1a).

*E. crus-galli* is an allohexaploid and therefore should be associated with ancient polyploidization events. We mapped *E. crus-galli* scaffolds to the diploid sorghum and foxtail millet (*S. italica*) genomes, and found that all chromosomes of sorghum and foxtail millet had three copies of *E. crus-galli* sequences aligned to them (Supplementary Fig. 3), consistent with *E. crus-galli* being a hexaploid. We calculated the transversion rate at the four-fold degenerate sites (4dTv) of paralogous and orthologous gene pairs in the *E. crus-galli* genome. The orthologous peaks of *Echinochloa-Oryza* and *-Sorghum* had a corresponding divergence of 4dTv ≈ 0.27 and 0.16 and dated to 46.7 and 27.9 Mya, respectively (Fig. 1b), which are consistent with the estimations based on the phylogenetic tree (Fig. 1a). The paralogous gene pairs based on syntenic blocks in the *E. crus-galli* genome showed one obvious large paralogous peak with 4dTv ≈ 0.042 dated the polyploidization event(s) for *E. crus-galli* speciation at ~ 7.8 Mya (Fig. 1b).

### Gene families associated with detoxification

**Fig. 2:**
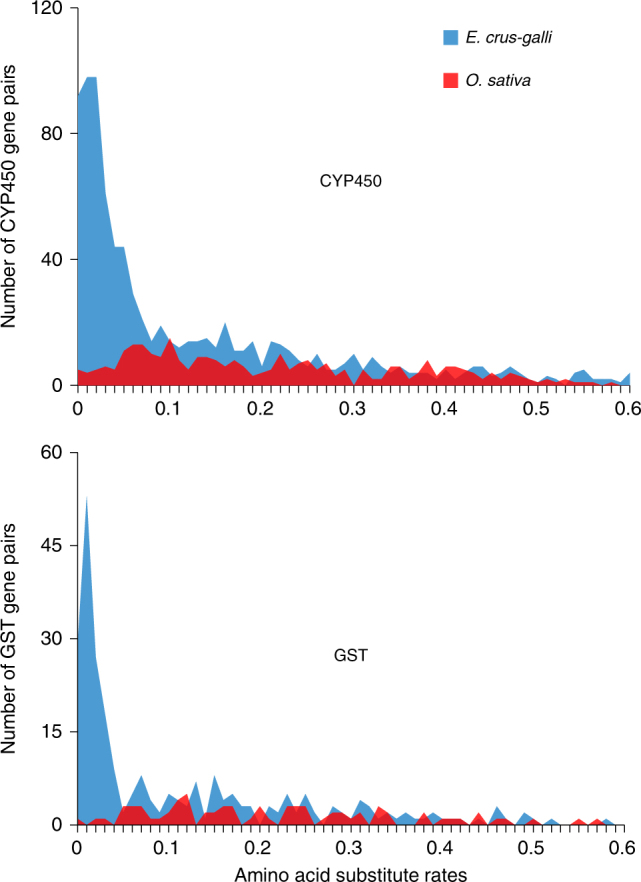
Gene families associated with detoxification in *E. crus-galli*. Distribution of amino acid substitute rates of *CYP450* and *GST* genes in *E. crus-galli* and *O. sativa* indicating a recent expansion of them in *E. crus-galli*

Gene families such as cytochrome P450 monooxygenase (CYP450) and glutathione S-transferase (GST) are commonly associated with the detoxification of allelopathic compounds or non-target-site resistance to synthetic herbicides in weeds^19^. Additionally, some members of the CYP450 family are often involved in the secondary metabolism biosynthesis^15^. In the *E. crus-galli* genome, a total of 917 CYP450 and 277 GST genes were identified, which is significantly higher than the numbers found in the genomes of the other five grasses (*O. sativa*, *B. distachyon*, *S. bicolor*, *S. italica*, *Z. mays*) used for comparison in this study and in Arabidopsis (249-354 CYP450 and 65-107 GST in a single genome). Enrichment analyses of Pfam domains showed that genes with the Pfam PF00067 (Cytochrome P450) and PF13417 (Glutathione S-transferase, N-terminal domain) are over-presented in *E. crus-galli* using other five grass species as background (PF00067: *P-*value = 3.52E-9; PF13417: *P*-value = 1.12E-3 both by hypergeometric test). Phylogenetic analysis indicates that the *E. crus-galli* genome contains more diverse *E. crus-galli*-specific branches in the two gene families relative to rice (CYP450 as an example shown in Supplementary Fig. 4). In addition, we investigated divergence times of CYP450 and GST genes in *E. crus-galli* and rice, respectively. A significantly large peak was observed at the range of very low amino acid substitution rates for CYP450 and GST genes in *E. crus-galli*, indicating a recent expansion which was likely caused by polyploidization events generating a large number of CYP450 and GST genes in *E. crus-galli* (Fig. 2). Transcriptomic profiles of *E. crus-galli* and rice plants involved in allelopathic interactions show that many genes from the two gene families are up-regulated (see below). These results suggest that the large repertoire of the CYP450 and GST genes may provide barnyardgrass enhanced detoxification ability under competition with nearby crop plants.

### Transcriptomic profiles of *E. crus-galli* allelopathy against rice

**Fig. 3:**
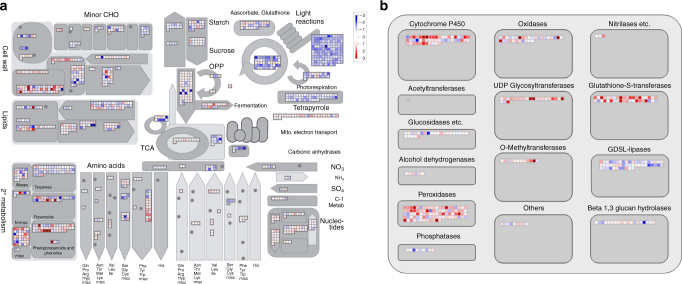
The allelopathic transcriptomic profiles of barnyardgrass against rice. **a** An overview for diverse pathways and **b** transcriptomic profile for large enzyme families visualized by Mapman. Color intensity corresponds to the expression fold change at log2 scale (red: up-regulated, blue: down-regulated)

To identify candidate genes involved in allelopathy of barnyardgrass against rice, the *E. crus-galli* line STB08 and a rice cultivar PI312777 (known to have a high allelopathic potential with barnyardgrass^20^) were grown separately (mono-cultured) or co-cultured (Supplementary Fig. 5). Transcriptome analysis was performed on the barnyardgrass plants following 3 h of cultivation. Using transcriptomes of mono-cultured plants as reference, a total of 4945 differentially expressed genes (DEGs), including 2534 up-regulated and 2411 down-regulated genes, were identified. Pathway enrichment analyses using Mapman software suggested that the significantly down-regulated genes in co-cultivated barnyardgrass are involved in photosynthesis and tetrapyrrole synthesis, suggesting rice plants growing with the barnyardgrass may have imposed an allelopathic effect on the weed by inhibiting its photosynthetic gene expression (Fig. 3a). In contrast, pathways associated with ‘cytochrome P450’, ‘brassinosteroid hormone metabolism’, ‘phenylpropanoid metabolism’ were enriched and the majority of the DEGs were up-regulated in the co-cultured barnyardgrass (Fig. 3b; Supplementary Data 1; Supplementary Fig. 6). Phenylpropanoid metabolism in particular generates an enormous array of secondary metabolites^21^. Brassinosteroids are phytohormones that have significant growth-promoting activity in plants and are involved in response to biotic and abiotic stresses^22^. The identified up-regulated DEGs in these enriched pathways may confer critical response pathways allowing barnyardgrass to protect itself against rice during their allelopathic interactions.

### The gene clusters for biosynthesis of DIMBOA in *E. crus-galli*

**Fig. 4:**
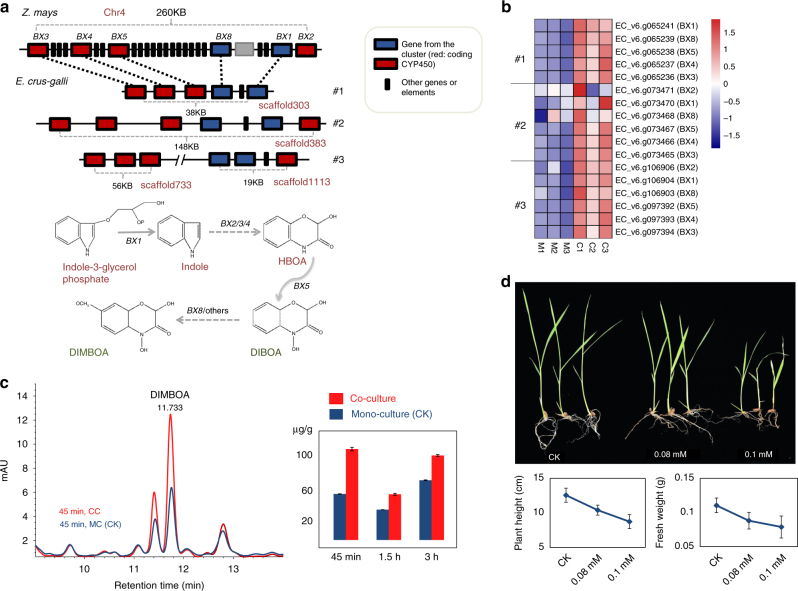
Characterization of biosynthetic gene clusters for DIMBOA in *E. crus-galli*. **a** Gene cluster for DIMBOA. Top, genomic synteny of the gene clusters for DIMBOA between barnyardgrass and maize. Bottom, the biosynthetic pathway of DIMBOA. **b** Expression changes of genes in the three copies of DIMBOA gene cluster in barnyardgrass during co-culture. Expression values were scaled for each gene. ‘M’ refers to mono-cultured experiments, and ‘C’ refers to experiments co-cultured with rice. **c** DIMBOA quantification in barnyardgrass. Left, selected HPLC ion chromatograms of root exudate extracts of barnyardgrass seedlings from mono-cultured (MC, as control) or co-cultured with rice (CC). Right, comparison of the DIMBOA concentrations in *E. crus-galli* from mono- and co-cultivated conditions at the specified time points. **d** Effect of DIMBOA treatment on the growth of rice seedlings. Top, phenotype of rice seedlings after one week treatment of 0.08 and 0.1 mM DIMBOA. Bottom, effects of DIMBOA treatment on plant height and fresh weight of rice seedlings

The gene cluster for DIMBOA production has been described only in maize so far, although the presence of DIMBOA has been reported in other upland plants (such as wheat and rye)^23^. Three copies of the DIMBOA gene cluster are found in the *E. crus-galli* STB08 genome each with perfect synteny with the maize genomic segment containing *BX1*-*5* and *BX8* (Fig. 4a). Our transcriptome data indicated that almost all copies of these genes (with the exception of BX2 and BX8 in copy 2) in *E. crus-galli* were significantly up-regulated in barnyardgrass co-cultured with rice compared to mono-cultured STB08 (Fig. 4b; Supplementary Data 2). To confirm that the induced expression of the DIMBOA biosynthetic gene cluster of barnyardgrass in response to co-cultivation with rice resulted in increased allelochemical production, we quantified the DIMBOA content in barnyardgrass. As shown in Fig. 4c, DIMBOA was significantly increased in barnyardgrass co-cultured with rice at all three time points (45 min, 1.5 and 3 h). As expected, no DIMBOA was detected in rice control plants.

Analysis of the effects of DIMBOA on rice growth showed that after DIMBOA treatment, rice plants showed a significant reduction in plant height and fresh weight. The inhibitory effects of DIMBOA were positively correlated DIMBOA concentration (Fig. 4d). Cumulatively these data suggest that the biosynthesis of DIMBOA by barnyardgrass is likely a significant mechanism in its competition with rice in the paddy field.

It is assumed that *E. crus-galli* (hexaploid) arises from the hybridization between tetraploid *E. oryzicola* (paternal donor) and an unknown diploid species (maternal donor)^8^. To determine the origins for the three copies of the DIMBOA gene clusters in *E. crus-galli* genome, we sequenced an *E. oryzicola* line (ZJU2, an estimated genome size of  ~ 1 Gb) to  ~ 30 × coverage (Supplementary Table 1 and Supplementary Fig. 7). Mapping results showed that scaffolds for two of the DIMBOA clusters (cluster copy 2 and 3) were well covered by the *E. oryzicola* reads, whereas the scaffold for cluster copy 1 could barely be mapped (Supplementary Fig. 7a–d). These results indicate that the three copies of the DIMBOA cluster in the hexaploid species *E. crus-galli* were likely generated from a hybridization event, during which two DIMBOA copies were contributed by the tetraploid *E. oryzicola* while the unknown diploid species ancestor contribute to another DIMBOA copy.

### The gene cluster for biosynthesis of momilactone A in *E. crus-galli*

**Fig. 5:**
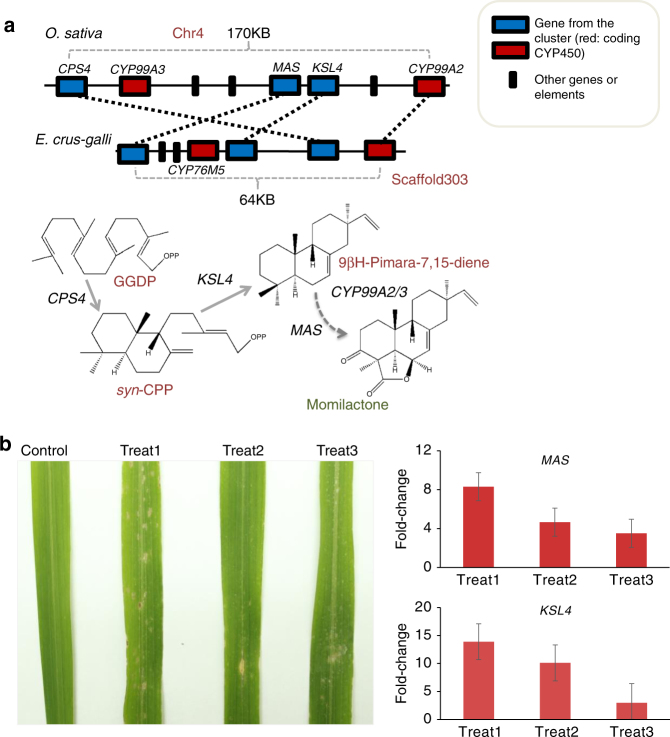
Characterization of the biosynthetic gene cluster for momilactone A in *E. crus-galli*. **a** Gene clusters responsible for biosynthesis of momilatone A in rice and barnyardgrass. Top, genomic synteny of the gene clusters for momilatone A between barnyardgrass and rice. Bottom, the biosynthetic pathway of momilatone A. Genomic positions of the gene clusters were adopted from Boycheva *et al*.^15^. **b** Phenotypes of leaves and expression changes of genes in the momilactone A cluster of *E. crus-galli* under *P. oryzae* infection. Left, phenotypes for three leaves under *P. oryzae* infection and one as control. Right, gene expression changes for the three infected leaves, three technical replicates were performed for each leave. Expression value of genes without *P. oryzae* infection (control) was normalized to be one

Interestingly, we also found a syntenic gene cluster in barnyardgrass corresponding to the gene cluster responsible for momilactone A biosynthesis in rice (Fig. 5a). Four adjoining genes in barnyardgrass were orthologous to four members of the gene cluster in rice despite some changes of gene order in the two genomes (Fig. 5a). However, the four genes in the *E. crus-galli* momilactone A biosynthesis gene cluster (only one copy found in the *E. crus-galli* genome) did not show any expression based on transcriptome analysis during barnyardgrass-rice co-cultivation. It has been previously reported that momilactones functions as both allelopathic and antimicrobial chemicals of rice against weeds and fungal pathogens, but can only exert little inhibition effect on the growth of seedlings of itself^24^. Besides, it has also been reported that momilactone A is significantly induced by blast (*P. oryzae*) infection of rice in paddy environments and serves as phytoalexin to enhance blast resistance in leaves^25^. Therefore we hypothesized that the momilactone A cluster in the *E. crus-galli* was also associated with fungal resistance through the antimicrobial activity of momilactone A in the paddy filed. To test this directly, we infected STB08 leaves with *P. oryzae* (Fig. 5b) and then examined the expression of the momilactone A synthase (*MAS*) and kaurene synthase-like (*KSL4*) genes in the momilactone A cluster using qRT-PCR. Our data show that the expression for the *MAS* and *KSL4* genes dramatically increased (in some cases exceeding 10-fold increases) after infection (Fig. 5b), indicating that the momilactone A gene cluster of *E. crus-galli* is activated by fungal stress and contributes to resistance to blast infection in paddy environment.

As the momilactone A gene cluster was previously only found in *Oryza* species^26^, the identification of a momilactone gene cluster in *E. crus-galli* that is distantly related to that found in *O. sativa* raises questions as to its evolutionary origins. Therefore, we conducted a phylogenetic analysis of the genes in the momilactone cluster from *E. crus-galli* and those from other grasses. Interestingly, our analysis showed that the *E. crus-galli KSL4*, *CPS4*, and *MAS* first clustered with their orthologs from *Oryza* species and then other cereals (Supplementary Fig. 8; Detailed gene labels see Supplementary Data 3). This clustering seems in contrast with the fact that *E. crus-galli* belongs to tribe Paniceae of the subfamily Panicoideae and near *S. italica* and *P. virgatum*, while *Oryza species* belong to the subfamily Oryzoideae (Fig. 1a; Supplementary Fig. 8a). The tree for *CYP450* gene (*CYP99A2*) in the momilactone A cluster is consistent with the species phylogeny (i.e., they clustered with their orthologs in *Setaria/Panicum* first) (Supplementary Fig. 8).

We also checked whether the genomic segment harboring the momilactone A cluster in *E. crus-galli* could be mapped by reads from *E. oryzicola*. Almost no reads could be mapped on the genes for the cluster (Supplementary Fig. 7e), suggesting that the momilactone A gene cluster in the *E. crus-galli* genome was not contributed by its paternal donor *E. oryzicola*.

## Discussion

We have generated the draft genome of the hexaploid species, *E. crus-galli*, one of the most notorious weeds known in modern crop fields. Our results provide new insights into the adaptive molecular mechanisms for their survival and invasiveness in the paddy field. Our data first shows that (1) *E. crus-galli* has evolved a large repertoire of detoxification-related genes (e.g., CYP450 and GST) and many of them are responsive during allelopathic interactions with rice; (2) *E. crus-galli* growing in paddy fields evolved multiple copies of the DIMBOA gene cluster which it uses in allelopathic interactions against rice and (3) also employs a momilactone A gene cluster against pathogenic fungi (and perhaps other biotic stresses) in the paddy field.

For polyploid species, sub-genomic sequence similarity is one of the challenges for genome assembly^27^, as it may be difficult to distinguish two or more similar sub-genomes in a species, which could possibly introduce mis-assembly and can influence following utility of the draft genome. Luckily, it seems that the divergence level among sub-genomes of allopolyploid species (e.g., tobacco^28^, cotton^29^, rapeseed^30^, and also *E. crus-galli* in this case) is high enough, and classical short sequencing data based assembly tools, such as SOAPdenovo and ALLPATHS-LG, can assemble their genomes well to a comparable assembly statistics (such as scaffold N50) to that of many diploid genomes. Currently, third generation sequencing (TGS) technologies have been comprehensively used in genome assembly. However, the polyploidy genome features could pose more challenges for utilization these data. It should be noted that the average sequencing error rate of TGS is still very high ( ~ 15%), which could be similar or even higher than the sequence differences between the sub-genomes of polyploidy species. Therefore, pre-correction step for TGS reads are quite necessary for using these data. In addition, we found that in our case, only less than 6 Gb corrected Pacbio data (efficiency < 20%) is left after correction by Canu, which may indicate that more raw data are needed for polyploidy genome sequencing projects.

Biosynthetic gene clusters responsible for known allelopathic compounds and phytoalexin (DIMBOA and momilactones) were found in the *E. crus-galli* genome, which have been identified in crops (such as maize and rice). Several benefits of gene clusters include improved efficiency in functionality, heredity, and survival compared with non-clustered genes functioning in the same pathway^15, 31^. These evolved functional gene clusters may imply one of the critical genetic bases for rapid environmental adaptability of the weed. For example, our results demonstrated that DIMBOA is an essential allelochemical employed by barnyardgrass to inhibit rice growth in paddy fields. *E. crus-galli* have evolved to form multiple copies of DIMBOA gene cluster, which should play a critical role in competition with rice and its invasiveness in paddy fields. Further, as to implications for rice breeding, it is crucial to find genes or loci responsible for DIMBOA resistance in rice genome, and rice cultivars with improved DIMBOA resistance may be produced through molecular breeding in the future.

The syntenic gene cluster for momilactone A has previously only been found in the rice genome. On the basis our transcriptome profiling analysis of baryardgrass and rice plants under co-cultivation, it does not appear that the momilactone A gene cluster in *E. crus-galli* is activated during these interactions. While the exact role(s) of the momilactone A cluster in *E. crus-galli* still requires further investigations, it appears that it may be involved in regulating biotic stress, primarily in response to infection of *P. oryzae*, a significant constraint on growth in rice paddies. The evolved momilactone A gene cluster in *E. crus-galli* could enhance its adaptability in paddy environment.

In investigation of the evolutionary history of momilactone A cluster, we only found one copy of momilactone A gene cluster in *E. crus-galli* genome, and it appears that the tetraploid paternal species *E. oryzicola* does not harbor this cluster or the related genes. Several reasonable explanations can be put forth to account for this observation. First, the unknown diploid *Echinochloa* species itself equips the momilactone A cluster, and contribute the cluster to *E. crus-galli* during hybridization with *E. oryzicola*. Alternatively, the diploid species does not harbor the cluster but has the related genes. After hybridization, the cluster formed in the *E. crus-galli* genome with genomic rearrangements of genes (*CPS4*, *KSL4*, *MAS*, and *CYP99A*) involved in the biosynthetic pathway. The unknown diploid *Echinochloa* species material is crucial for better understanding of the evolutionary mystery of the momilactone A gene cluster, and the environmental adaptation of *Echinochloa* weeds. Our phylogenetic analyses showed that genes of *E. crus-galli* in the momilactone A gene cluster seems closer to *O. sativa* than other phylogenetically close species (e.g., *B. distachyon*, *Z. latifolia* or even *O. brachyantha*). The unexpected phylogenetic distributions suggested that complex processes (e.g., multiple gene duplications followed by different losses, or even horizontal gene transfer) could be involved in the evolution of the genes (*CPS4*, *KSL4*, *MAS*) and the formation of the momilactone gene cluster in *E. crus-galli*, which need further investigation in future.

## Methods

### Genome sequencing and assembly

The *E. crus-galli* line STB08, collected from rice paddy fields in the lower Yangtze River region of China (30°17′ N, 119°57′ E). Genomic DNA was extracted from young leaves of *E. crus-galli* (STB08) plants using the CTAB method. RNase A and proteinase K were used to remove RNA and protein contamination, respectively. Paired-end and mate-pair Illumina genomic DNA libraries with different insertion sizes (160 bp–20 Kb) were constructed following the manufacturer’s instructions (Illumina, USA). The libraries were sequenced on an Illumina HiSeq 2000 system. Meanwhile, two Pacbio RS II P4-C2 libraries of STB08 were constructed following the manufacturer’s instructions and 48 SMRT cells (32.9 Gb) were sequenced. Raw reads were processed by removing PCR duplicates, low-quality reads, adaptor sequences and contaminated reads with bacterial or viral origin. In addition, we used Lighter software^32^ to correct the reads from each library based on the *K*-mer frequency. The resulting clean reads were assembled into contigs and scaffolds using SOAPdenovo2^33^ with the default settings. The assembled sequences were further scaffolded with OPERA-LG v2.0.5^34^, and gaps within the scaffolds were filled using GapCloser v1.1^33^. Redundans^35^ was used to filter redundant sequences due to heterozygosity. Raw Pacbio RS long reads were corrected by Canu^36^, and then were used to fill the gaps by PBjelly v14.1with the default settings^37^. Detailed assembly pipeline and statistics are shown in Supplementary Data 4, respectively. Both CEGMA^38^ and BUSCO v2^39^ were used to evaluate the completeness of assembled genome. Fosmid libraries were prepared following the manual of CopyControl Fosmid Library Production Kit (Epicentre, Madison, WI). In addition, 74 publicly available *E. crus-galli* EST sequences and 156,757 transcripts generated by this study were aligned to the assembled genome by GMAP^40^ for validation. For *E. oryzicola* line ZJU2 (collected from 23°31′N, 111°43′E), paired-end DNA libraries (800 bp) were prepared and sequenced by HiSeq2500. Clean reads were mapped to the assembled *E. crus-galli* genome by Bowtie2^41^ and the mapping result was visualized using inGAP^42^.

### Genome gene and repeat annotation

We constructed an *E. crus-galli* repeat library using RepeatModeler v1.0.5^43^ with the default parameters. Two complementary programs (RECON and RepeatScout) were configured in RepeatModeler and were used for identification of repeat family sequences in the genome. The resulting *E. crus-galli* repeat library was further used to run RepeatMasker v3.1.2^43^ for the whole genome repeat annotation.

A hybrid strategy combining ab initio predictions, homologous gene evidence and transcriptomic support (RNA-seq) was applied in gene prediction. Three ab initio gene finders, GeneMark.hmm^44^, Fgenesh^45^, and Augustus^46^ were used. Protein sequences of three closely related species (*Z. mays*, *S. bicolor*, *S. italica*) were aligned to the assembled *E. crus-galli* genome using Spaln2^47^ to get evidences of the gene structure. RNA-seq data generated from the whole plant of *E. crus-galli* were used for gene annotation. The reads were *de novo* assembled by Trinity^48^ into raw transcripts. The Seqclean utility implemented in PASA^49^ was applied to identify evidence of polyadenylation, strip the poly-As, trim vectors, and to discard low-quality sequences. The remaining transcripts were aligned to the *E. crus-galli* genome using GMAP. The valid transcript alignments were clustered and further assembled by PASA based on genomic location and then the transcriptome-based consensus gene models were generated. All gene structures predicted by the above procedures were integrated into consensus gene models by EVM^50^. The predicted genes were then checked manually. The final set of genes were determined using the following criteria: (1) Partial gene models without start or stop codons were removed; (2) No ‘N’s residing in the coding sequences (CDS); (3) Sequences homologous with the Repbase (E-value ≤ 1e-5, identity ≥ 30%, coverage ≥ 30% and minimum matching length ≥ 30 aa) were removed.

The predicted *E. crus-galli* genes were aligned against non-redundant green plant protein databases in Swiss-Prot and NR protein databases for functional annotation (BLASTP, E-value ≤ 1e-5). Protein domains were predicted by comparing the sequences against various domain databases, including Pfam, TIGRFAM, ProDom, and SMART using InterProScan v5^51^. Gene ontology (GO) terms for each gene were assigned based on corresponding InterPro entries. Non-coding RNAs were predicted by the Infernal program using default parameters^52^.

### Phylogenetic tree and genomic synteny

We identified paralogs and orthologs using OrthoMCL v1.4 with the default settings (BLASTP E-value ≤ 1e-5 and MCL inflation parameter of 1.5)^53^. The protein sequence sets from six sequenced plants (*B. distachyon*, *O. sativa*, *S. bicolor*, *S. italica*, *Z. mays*, *A. thaliana*; Phytozome v9.0) and *E. crus-galli* were used to perform gene family identification. The protein sequences of single-copy genes among the seven species identified by OrthoMCL were aligned using MAFFT^54^. After the Gblocks^55^ alignment optimization, single-copy genes with aligned protein sequences longer than 1000 amino acids (aa) were chosen for phylogenetic tree construction and divergence time estimation. Species divergence times were estimated using an uncorrelated relaxed clock in BEAST v1.7.5 with *A. thaliana* as an outgroup^56^. Monophyletic constraints were imposed for the nodes that were used to calibrate the evolutionary rates (Blosum62 and an uncorrelated exponential relaxed model). We used a Yule speciation process, which specifies a constant rate of species divergence. Normal priors were used for monocot-dicot split time (mean: 150.0 Mya, std dev: 4.0) and for *O. sativa*-*Z. mays* split time (mean: 50.0 Mya, std dev: 4.0). The MCMC chains in BEAST were run for 10,000,000 generation sampling every 1,000 steps. Convergence between the runs and the amount of burn-in (throwing away some iterations at the beginning of an MCMC run) was determined using Tracer v1.5^57^, which was used to assess the effective sample size and to check the consistency of the results. The tree was drawn with FigTree^58^. For genomic synteny, we first used BLASTP (E-value ≤ 10-7) to align the *E. crus-galli* protein dataset to that of *S. bicolor*, *S. italica* and itself. The alignments were then subjected to DAGchainer^59^ to determine syntenic blocks. The 4dTv of each syntenic block was calculated by an in-house Perl script.

To build phylogenetic trees for the genes of momilactone A gene clusters, ten grass species (including *O. sativa*, *O. punctata*, *O. brachyantha*, *Zizania latifolia*, *Brachypodium distachyon*, *S. bicolor*, *Z. mays*, *Panicum virgatum*, and *Setaria italic, E. crus-galli*) were involved and *Arabidopsis thaliana* was used as outgroup. BLASTP was used to scan homologous genes to rice genes of the cluster in the protein dataset (E-value thresholds: MAS: 1e-80; KSL4, CPS4, and CYP99A2: 1e-100). Raxml v8^60^ was applied with the parameters ‘-m PROTGAMMAAUTO –auto-prot = bic’ to automatically select the best protein model for tree construction^60^. Each tree was constructed with 100 bootstraps.

### Investigations for P450 and GST

Members of the CYP450 (PF00067) and GST (PF13417, PF13410, PF13409, PF00043, and PF02798) gene families were identified by InterProScan v5^51^. The phylogenetic tree was constructed by Fasttree^61^ with protein sequence alignments by MAFFT v7.2^54^. For divergence estimation of *CYP450* and *GST* genes, phylogenetic trees of *CYP450* and *GST* genes in *E. crus-galli* and *O. sativa* were constructed by Fasttree^61^ with an Arabidopsis gene (*CYP450*: AT1G75130.1; *GST*: AT1G59670.1) as outgroup, respectively. The substitution distances are measured by MEGA v5.2^62^. On the basis of identified Pfam information for all genes of five grass species (*O. sativa*, *B. distachyon*, *S. bicolor*, *S. italica*, *Z. mays*) and *E. crus-galli* based on InterProScan v5^51^, enrichment analysis was conducted to examine whether *CYP450* and *GST* genes are over-presented in the *E. crus-galli* genome by hypergeometric test.

### Genomic and transcriptomic investigations for allelopathy

We searched barnyardgrass orthologs in known biosynthesis gene clusters^15^ to identify candidate gene clusters in the *E. crus-galli* genome that are involved in the allelopathic interaction.

The relay seeding in agar (RSA) method^63^ with a few modifications was used to investigate the allelopathic interactions between barnyardgrass (STB08) and rice (PI312777). Ten germinated PI312777 seeds were first transferred to a plastic tissue culture box (10 cm in base diameter) filled with 50 ml of medium containing 0.5% agar, and arranged in three rows with a 3-4-3 pattern (Supplementary Fig. 5). Germinated STB08 seeds were first transferred to a Petri dish with sterile water. Five days later, 10 germinated STB08 seeds were transferred to the tissue culture box containing germinated PI312777 seeds with a pattern of five STB08 seeds between two rows of PI312777 seeds (Supplementary Fig. 5). The PI312777 and STB08 seedlings were co-cultured in a SAFE incubator (Ningbo, China) kept at 75% relative humidity. Mono-cultured STB08 seedlings (i.e., seedlings growing alone, under the same conditions) were used as controls. The entire co- and mono-cultured STB08 seedlings were collected at 3 h and RNA extracted for RNA-Seq analysis. A total of three biological replicates were collected for each condition.

Illumina RNA-Seq libraries were prepared and sequenced on a HiSeq4000 system following the manufacturer’s instructions. The raw paired-end reads were first filtered into clean data using NGSQCtookit v2.3.3 with default settings^64^. The cleaned reads were then aligned to the *E. crus-galli* genome assembly using Tophat v2.0.9^65^. After alignment, the count of mapped reads from each sample was derived and normalized to fragments per kilobase of exon per million fragments mapped for each predicted transcript using the Cufflinks package^66^. The resulting alignment files were then supplied to Cuffdiff in the Cufflinks package for differential expression analysis.

The allelopathic transcriptomic profile of our experiment was investigated using the Mapman software^67^. All *E. crus-galli* genes were first submitted to the online functional annotation sever called Mercator^68^. The log2 ratio of fold change (co-culture/mono-culture) was calculated for each significantly differentially expressed gene. The resulting files were loaded into Mapman to visualize the transcriptomic profile and perform the pathway enrichment study.

### Experiments for DIMBOA measurement

For DIMBOA measurement, mono- (six plants in a tissue culture box) or co-cultivated (three *Echinochloa* and three rice plants in a box) hydroponic *Echinochloa* weeds (STB08) and rice (PI312777) seedlings at the first-leaf stage were transplanted to deionized water for 45 min, 1.5 and 3 h. Both mono- and co-cultivated *Echinochloa* and rice plants (whole plant) were then analyzed by HPLC after freeze-drying.

### Experiments for *P. oryzae* infection

*Pyricularia oryzae* strains were cultured on agar plates containing complete medium for 10 days and conidia were harvested and re-suspended in 0.2% (w/v) gelatin solution. About 10^5^ conidia per ml were sprayed evenly onto *E. crus-galli* plants at the three-leaf stage using an artist’s airbrush (Badger Co., Illinois) with the 0.2% (w/v) gelatin solution as control^69^. The inoculated plants were placed in a dew chamber at 22 °C for 48 h in the dark and then transferred in a growth chamber under a photoperiod of 12 h for 7 days. Leaves with lesion was used for examination of gene expression by qRT-PCR. The relative value for the expression level of *MAS* and *KSL4* genes was calculated by the ${2^{ - \Delta \Delta {C_{\rm{T}}}}}$ method using the Tubulin gene as an internal control. Primers for qRT-PCR were given in Supplementary Table 7.

### Data availability

All raw DNA sequencing reads (Illumina and PacBio data) for *E. crus-galli de novo* assembly, sequencing data for *E. oryzicola*, and RNA-Seq data for the gene prediction and allelopathic experiments have been deposited under NCBI BioProject PRJNA268892. The SRA accession numbers are SRR5920284-SRR5920293, SRR5902661, SRR5903813-SRR5903830, and SRR5903559-SRR5903564. The *E. crus-galli* genome assembly and the annotated genes are accessible at http://ibi.zju.edu.cn/RiceWeedomes/Echinochloa/, and the assembly is also available at ENA (European Nucleotide Archive) under assembly accession GCA_900205405. All relevant data contained within the paper are available from the corresponding author on request.

## Electronic supplementary material

Supplementary Information

Description of Additional Supplementary Files

Supplementary Data 1

Supplementary Data 2

Supplementary Data 3

Supplementary Data 4
