# Supplementary material for: Echinochloa crus-galli genome analysis provides insight into its adaptation and invasiveness as a weed
Source: Nat Commun. 2017 Oct 18;8:1031. doi: 10.1038/s41467-017-01067-5 (PMC5647321; doi:10.1038/s41467-017-01067-5)
Supplement: Supplementary file 1 — Supplementary Information [file 41467_2017_1067_MOESM1_ESM.pdf]

Supplementary Information

Supplementary Figures

a

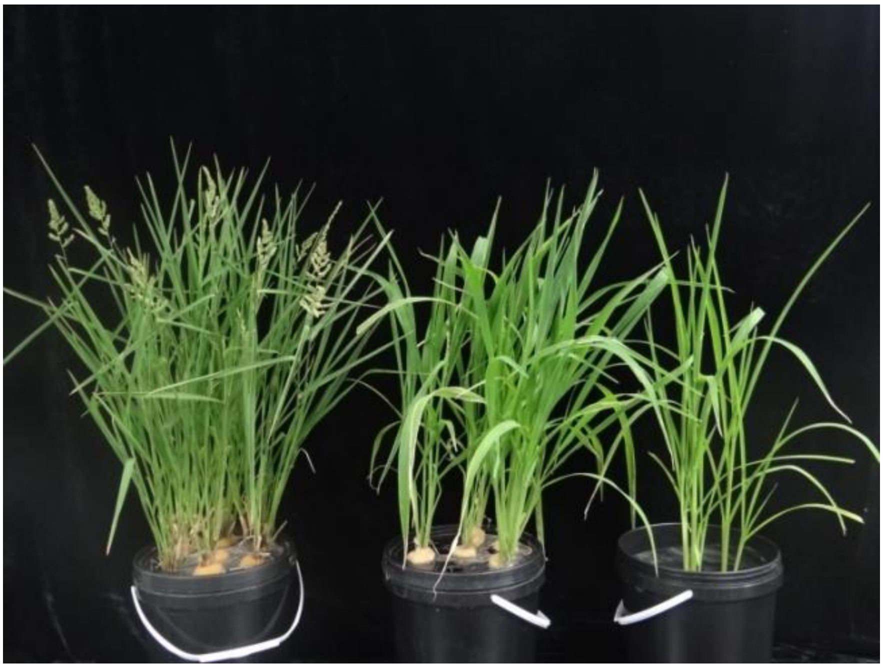

b

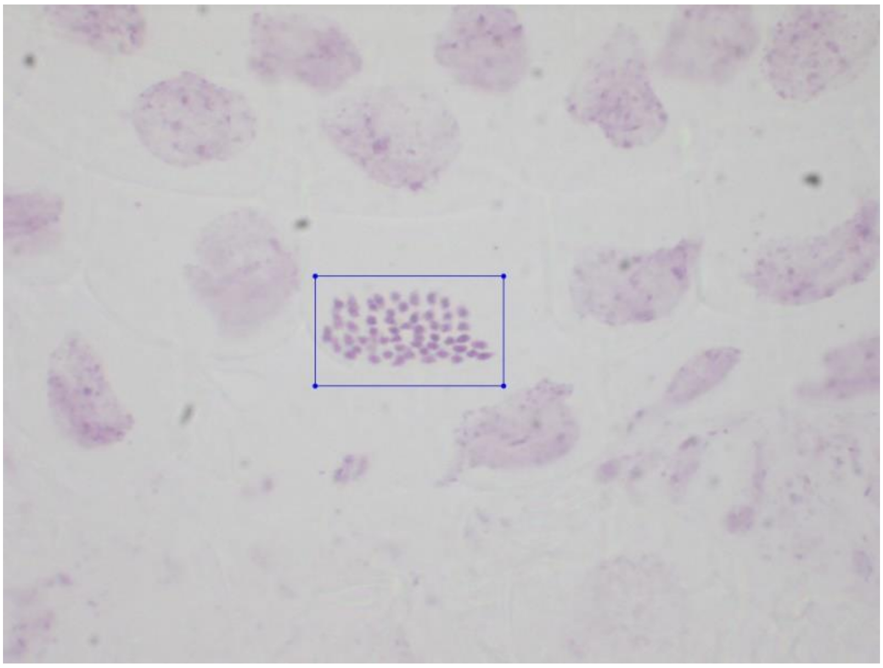

Supplementary Figure 1. Phenotypes and basic chromosome number of *E. crus-galli* (STB08). (a) Photographic image of *E. crus-galli* line STB08 showing that it resembles rice in morphology. **(b)** Determination of chromosome number using acetocarmine staining showing that the chromosome number of *E. crus-galli* line STB08 is 2n=54.

a

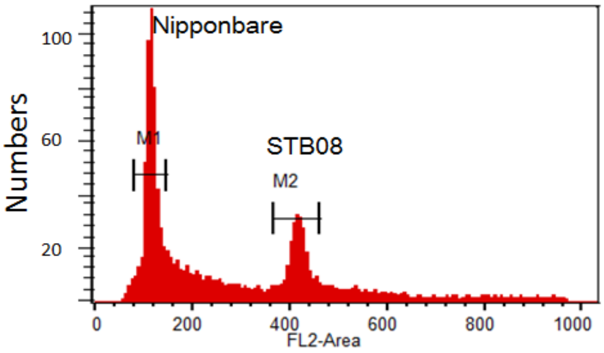

b

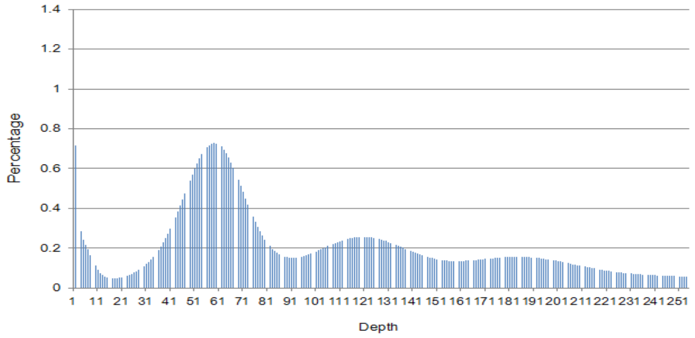

c

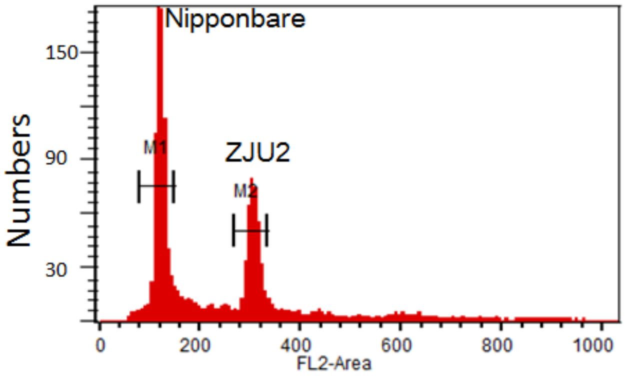

c

Supplementary Figure 2. Genome size estimation of *E. crus-galli* and *E. oryzicola*. (a) Genome size estimation of *E. crus-galli* line STB08 by flow cytometry using the rice cultivar Nipponbare as an internal reference. **(b)** Distribution of 17-mer based on Illumina reads. The volume of *K*-mer is plotted against the frequency at which they occur. The total number of *K*-mers is 83,083,125,203 and the volume peak is 59. The genome size of 1,382 Mb was estimated as by total *K*-mer number - *K*_unique)/(volume peak). **(c)** Genome size estimation of *E. oryzicola* line ZJU2 by flow cytometry using the rice cultivar Nipponbare as an internal reference.

a

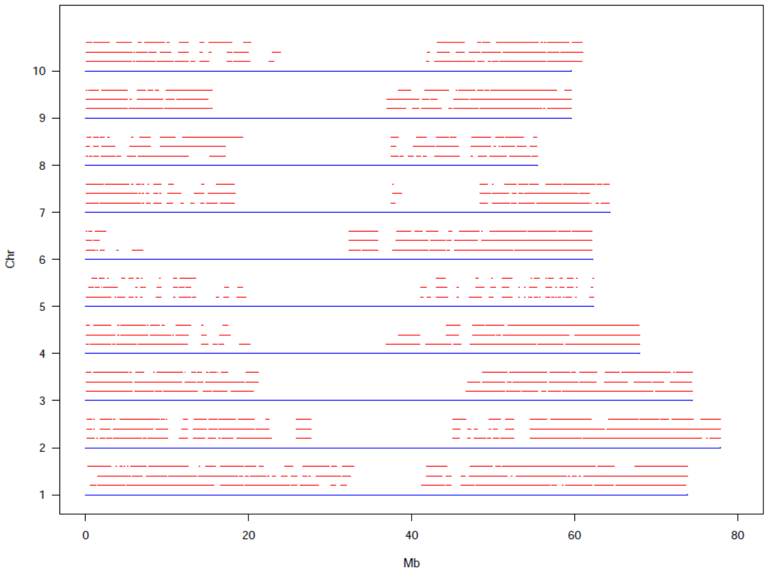

b

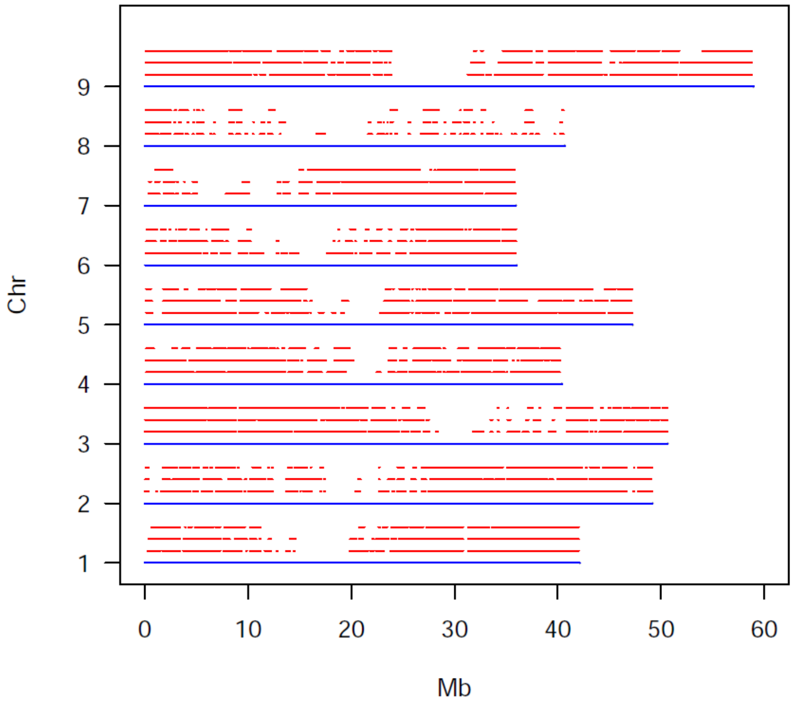

Supplementary Figure 3. Genomic synteny between *E. crus-galli* and two related species, *S. bicolor* and *S. italica*. *E. crus-galli* scaffolds were uniquely mapped to the (**a**) diploid *S. bicolor* and (**b**) *S. italica* genomes. The blue lines represent the *S. bicolor* and *S. italica* chromosomes and the red segments stand for *E. crus-galli* scaffolds.

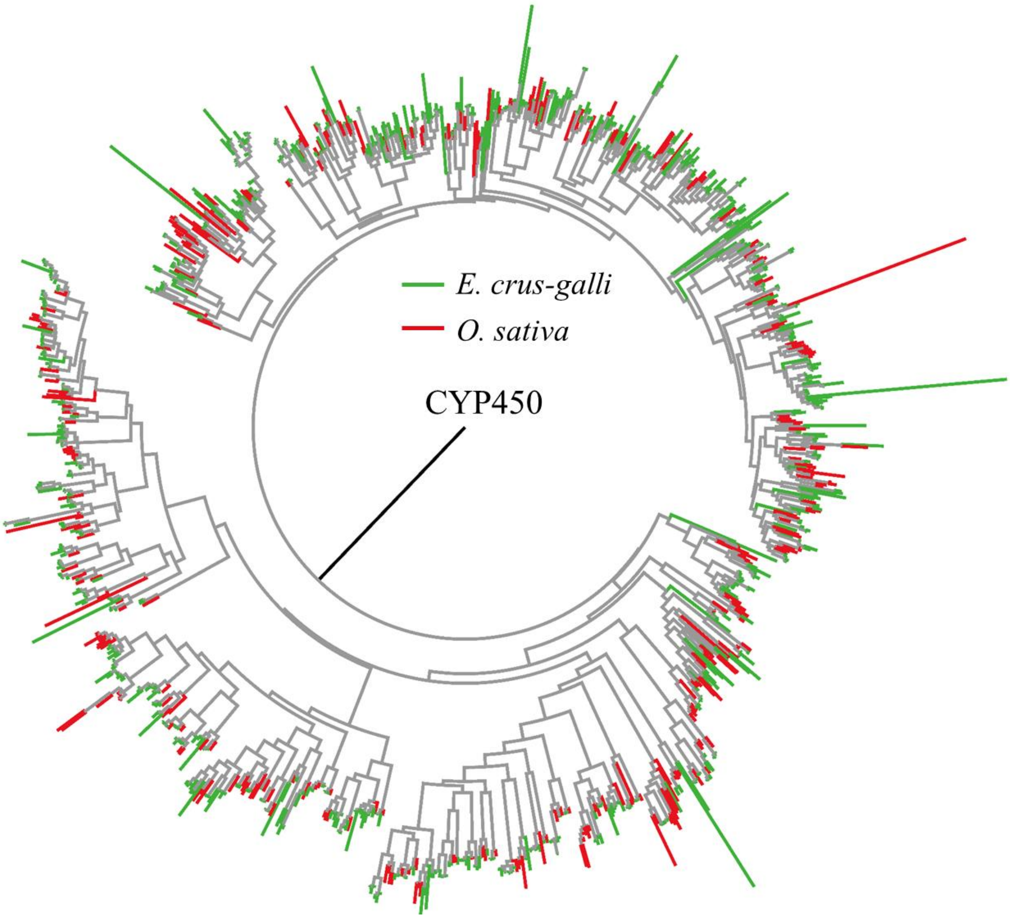

Supplementary Figure 4. Phylogenetic relationship of *CYP450* gene family members in *E. crus-galli* and *O. sativa*. The branches for *O. sativa* and *E. crus-galli* are colored as red and green, respectively.

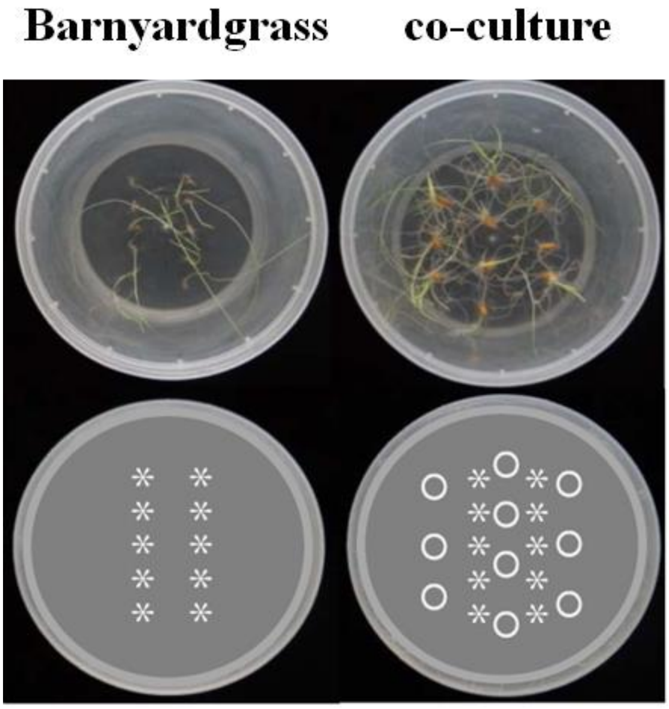

Supplementary Figure 5. Experimental design for transcriptome investigation of allelopathic interactions between *E. crus-galli* and *O. sativa*. Top row: growth of barnyardgrass alone (left) and in in co-cultivation with rice seedlings (right). Bottom row, diagrams showing the seedling layout of barnyard grass (asterics) and rice (circles) in the plates above. Three biological replicates of the experiment were performed.

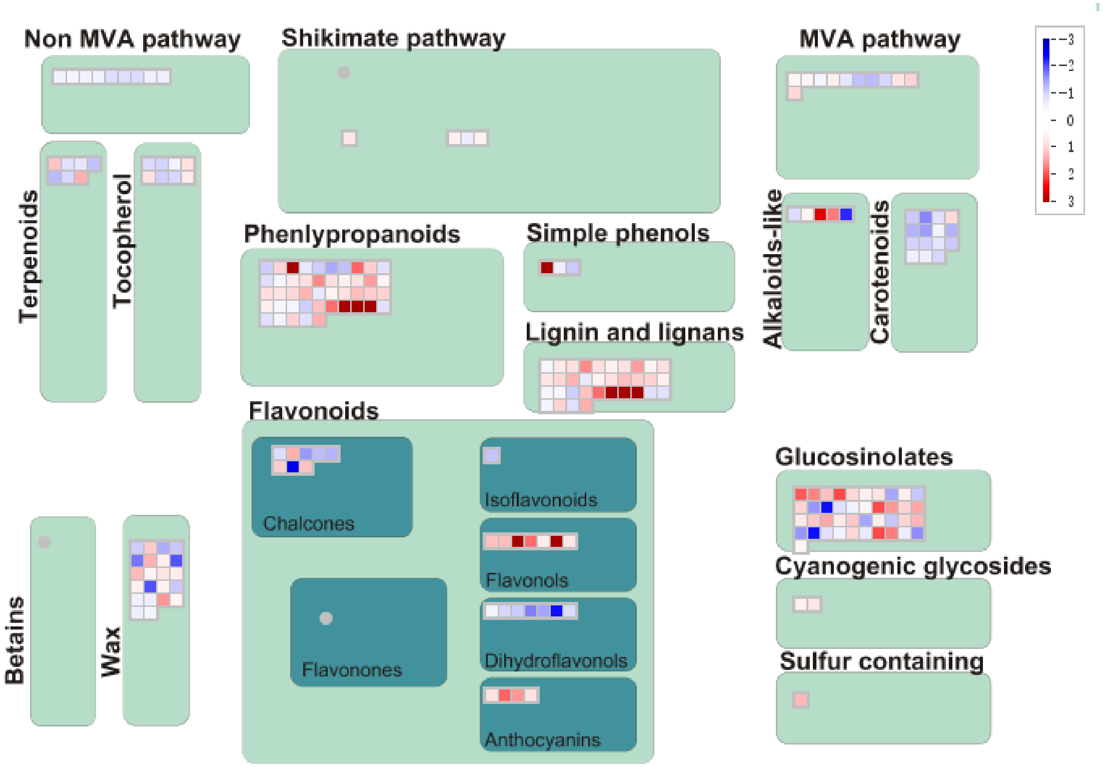

Supplementary Figure 6. Mapman visualization of allelopathic transcriptomic profile of barnyardgrass against rice. Shown in the figure are the secondary metabolism transcriptomic profiles of various pathways. Color intensity corresponds to the expression fold change at log2 scale (red: up-regulated; blue: down-regulated).

a. **gene cluster for DIMBOA (copy 1)**

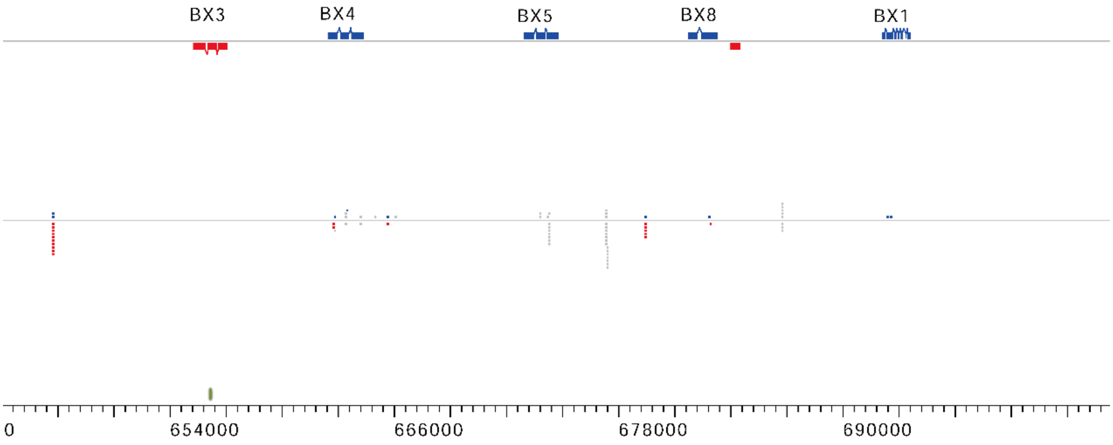

b. **gene cluster for DIMBOA (copy 2)**

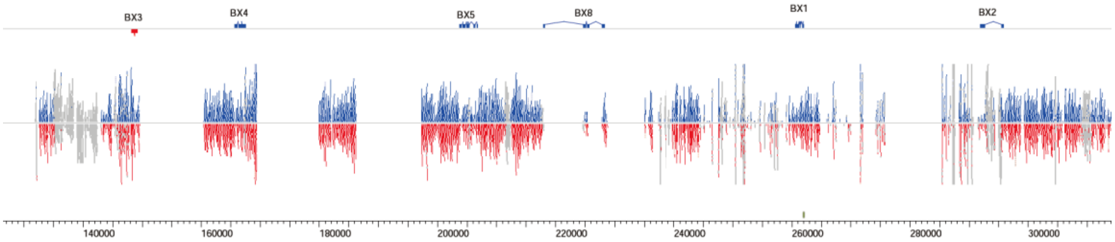

c. **gene cluster for DIMBOA (one part of copy 3)**

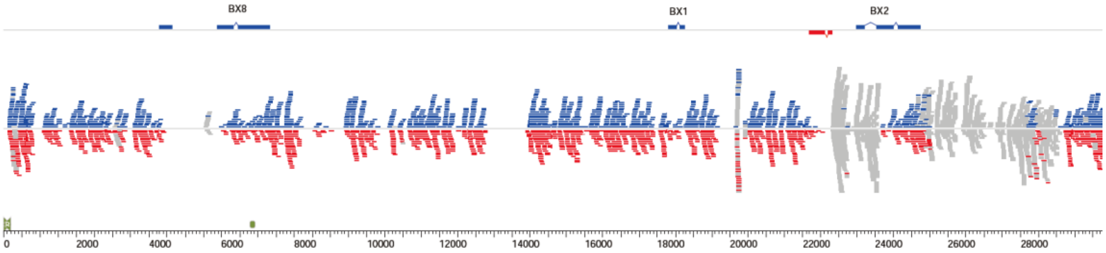

d. **gene cluster for DIMBOA (the other part of copy 3)**

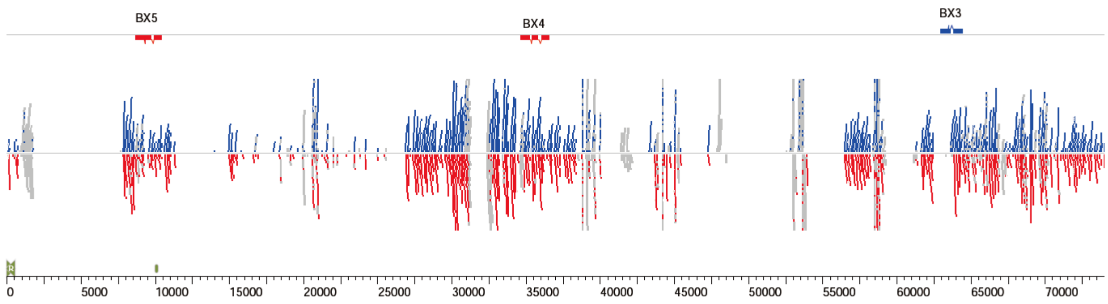

e. **gene cluster for momilactone A**

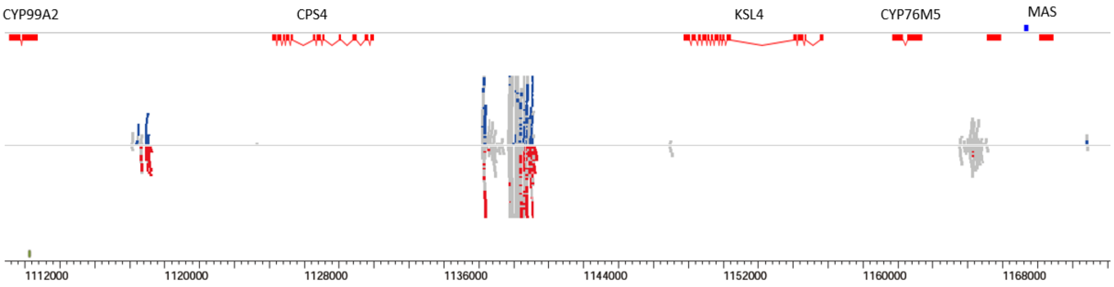

Supplementary Figure 7. The mapping results of *E. oryzicola* (ZJU2) against *E. crus-galli* for the gene clusters of momilactone A and DIMBOA. For each panel (a-e), gene cluster from *E. crus-galli* and mapped reads from ZJU2 are indicated in top and bottom, respectively. Mapped reads with different directions are indicated by red and blue, with those of repetitive sequences indicated by grey.

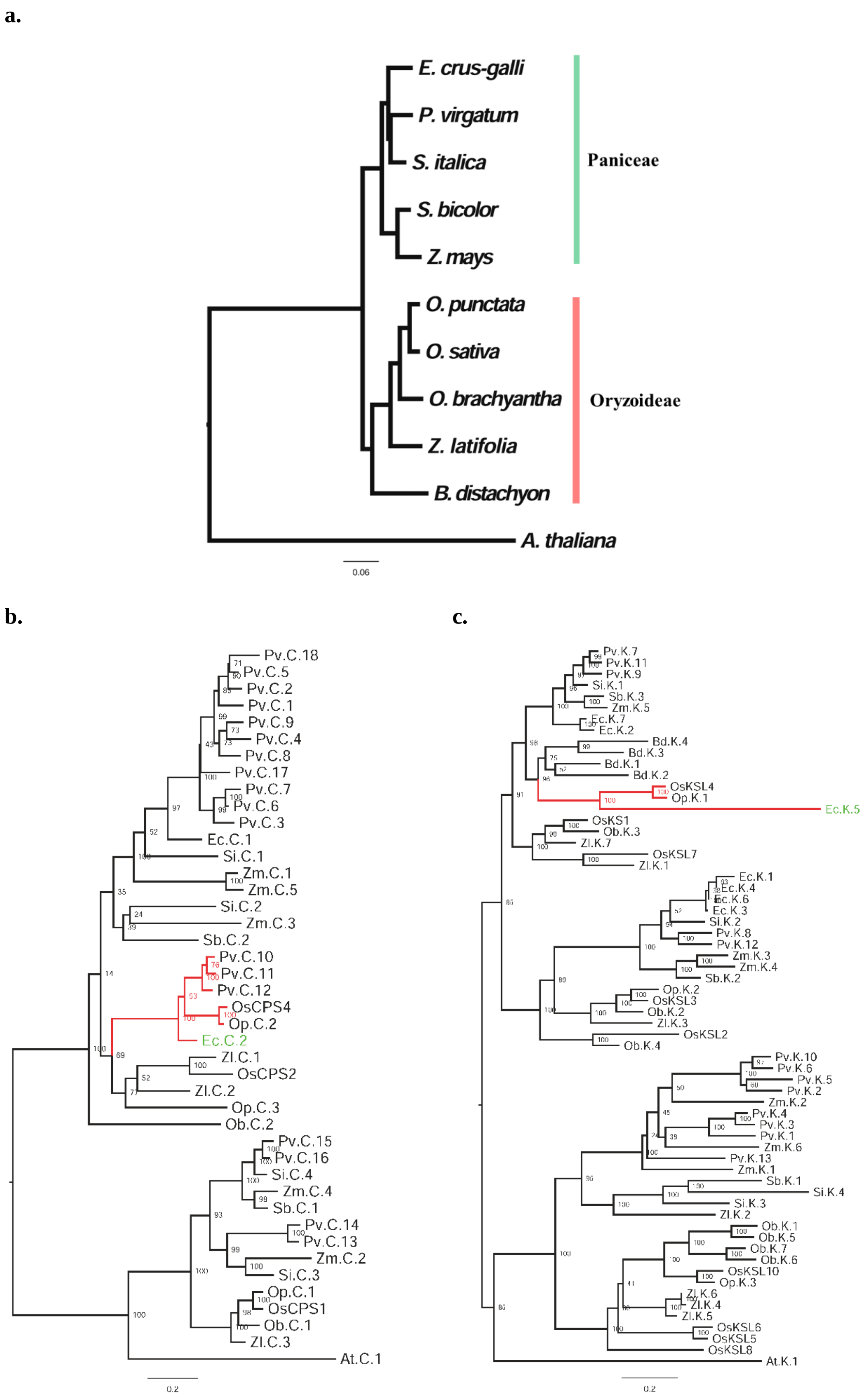

a.
E. crus-galli
P. virgatum
S. italica
S. bicolor
Z. mays
Paniceae
O. punctata
O. sativa
O. brachyantha
Z. latifolia
B. distachyon
Oryzoideae
A. thaliana
0.06
b.
Pv.C.18
Pv.C.5
Pv.C.2
Pv.C.1
Pv.C.9
Pv.C.4
Pv.C.8
Pv.C.17
Pv.C.7
Pv.C.6
Pv.C.3
Ec.C.1
Si.C.1
Zm.C.1
Zm.C.5
Si.C.2
Zm.C.3
Sb.C.2
Pv.C.10
Pv.C.11
Pv.C.12
OsCPS4
Op.C.2
Ec.C.2
Zl.C.1
OsCPS2
Zl.C.2
Op.C.3
Ob.C.2
Pv.C.15
Pv.C.16
Si.C.4
Zm.C.4
Sb.C.1
Pv.C.14
Pv.C.13
Zm.C.2
Si.C.3
Op.C.1
OsCPS1
Ob.C.1
Zl.C.3
At.C.1
0.2
c.
Pv.K.7
Pv.K.11
Pv.K.9
Si.K.1
Sb.K.3
Zm.K.5
Ec.K.7
Ec.K.2
Bd.K.4
Bd.K.3
Bd.K.1
Bd.K.2
OsKSL4
Op.K.1
Ec.K.5
OsKS1
Ob.K.3
Zl.K.7
OsKSL7
Zl.K.1
Ec.K.1
Ec.K.4
Ec.K.6
Ec.K.3
Si.K.2
Pv.K.8
Pv.K.12
Zm.K.3
Zm.K.4
Sb.K.2
Op.K.2
OsKSL3
Ob.K.2
Zl.K.3
OsKSL2
Ob.K.4
Pv.K.10
Pv.K.6
Pv.K.5
Pv.K.2
Zm.K.2
Pv.K.4
Pv.K.3
Pv.K.1
Zm.K.6
Pv.K.13
Zm.K.1
Sb.K.1
Si.K.4
Si.K.3
Zl.K.2
Ob.K.1
Ob.K.5
Ob.K.7
Ob.K.6
OsKSL10
Op.K.3
Zl.K.6
Zl.K.4
Zl.K.5
OsKSL6
OsKSL5
OsKSL8
At.K.1
0.2

d.

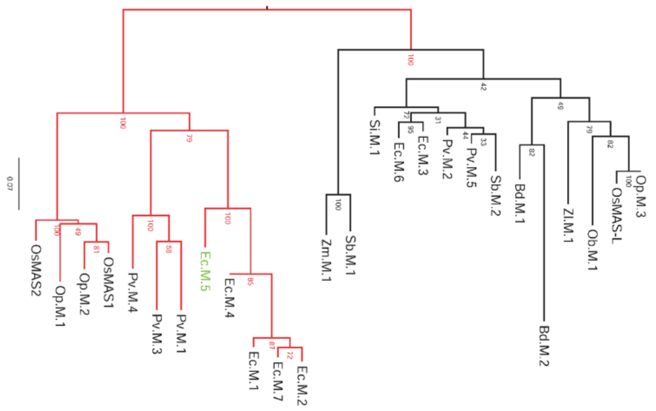

e.

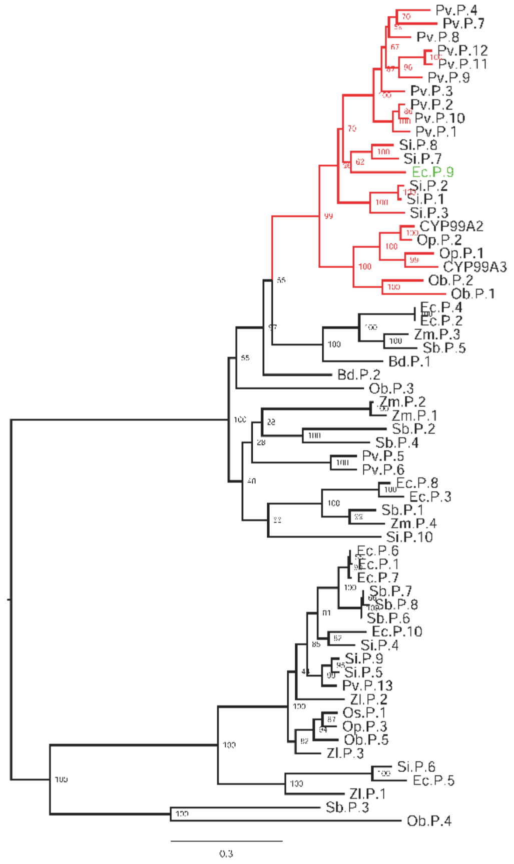

Supplementary Figure 8. Detailed phylogenetic analysis of gene families involved in the biosynthesis of momilactone A in the grasses. (a) Phylogenetic tree for nine members of the grass family. Phylogenetic trees of the (b) *CPS4*, (c) *KSL4*, (d) MAS, and (e) P450 gene found in the gene clusters responsible for momilactone A biosynthesis in *E. crus-galli* and nine members of the grass family. Ec.C.2, Ec.K.5, Ec.M.5, and Ec.P.9 are syntenic orthologs of the rice *CPS4*, *KSL4*, MAS, and CYP99A2 genes, respectively. The numbers shown at nodes indicate bootstrap values. The names of orthologous genes from *A. thaliana*, *B. distachyon*, *E. crus-galli*, *O. sativa*, *O. punctata*, *O. brachyantha*, *Z. latifolia*, *P. virgatum*, *S. bicolor*, *S. italica*, and *Z. mays* are indicated by the suffix “At”, “Bd”, “Ec”, “Os”, “Op”, “Ob”, “Zl”, “Pv”, “Sb”, “Si” and “Zm”, respectively. The full gene IDs are listed in Supplementary Table 9.

Supplementary Tables

Supplementary Table 1. Summary of genome sequencing of *E. crus-galli* (STB08) and *E. oryzicola* (ZJU2).

STB08

Pair-end libraries	Insert size (bp)	Reads number	Reads length (bp)	Total bases (Gb)	Genome coverage (X)
160	160	440,429,122	100	44	31.4
300	300	196,000,076	100	19.6	14
500	500	312,896,950	90	28.2	20.1
500_1	500	104,938,914	100	10.5	7.5
2k	2,000	122,259,494	100	12.2	8.7
2k_2	2,000	117,315,348	100	11.7	8.4
5k	5,000	130,108,084	100	13	9.3
10k	10,000	173,189,822	100	17.3	12.4
20k	20,000	406,934,730	125	50.9	36.4
Total Illumina data		2,004,072,540		207.4	148.2
Pacbio RSII	P4-C2		~1700	32.9	23.5

ZJU2

Pair-end libraries	Insert size (bp)	Reads number	Reads length	Total bases (Gb)	Genome coverage (X)
800	800	258,225,620	125	32.3	32.4

Supplementary Table 2. Summary of alignments of the fosmid contig sequences and genomic scaffolds of *E. crus-galli*.

Clone_ID	Clone_length (bp)[a]	Scaffold	Alignment_length (bp)[b]	Coverage[c]	Identity[d]
Fosmid1	20,697	Scaffold251	19,014	91.9%	99.5%
Fosmid2	20,875	scaffold134	20,218	96.9%	99.8%
Fosmid3	15,807	scaffold404	14,402	91.1%	99.1%
Fosmid4	16,576	scaffold466	16,512	99.6%	99.8%
Fosmid5	15,048	scaffold180	14,880	98.9%	99.6%

a The length of the final clone assembly

b The length of alignment of clone and scaffold sequences by the mVista

c Coverage = aligment length/clone length

d Identity refers to the percentage of matched bases in the aligned sequences

Supplementary Table 3. Statistics of the completeness of assembled *E. crus-galli* genome based on CEGMA and BUCSO.

(a) CEGMA result

	**Numbers of CEGs[a]**	**Mapped proteins**	**Completeness (%)**	**Total[c]**	**Average[d]**	**Ortho(%)[e]**
Complete[b]	248	229	92.34	855	3.73	96.51
Group 1	66	61	92.42	207	3.39	95.08
Group 2	56	50	89.29	194	3.88	96
Group 3	61	57	93.44	206	3.61	94.74
Group 4	65	61	93.85	248	4.07	100
Partial[b]	248	239	96.37	988	4.13	97.91
Group 1	66	64	96.97	247	3.86	98.44
Group 2	56	54	96.43	221	4.09	96.3
Group 3	61	60	98.36	251	4.18	96.67
Group 4	65	61	93.85	269	4.41	100

a The CEGs database contains groups of genes from six species (*Homo sapiens*, *Drosophila melanogaster*, *Arabidopsis thaliana*, *Caenorhabditis elegans*, *Saccharomyces cerevisiae* and *Schizosaccharomyces pombe*). The CEGs were classed as four groups based on the conservation.

b Complete and partial refer to the length of alignments.

c Total number of CEGs present including putative orthologs.

d Average number of orthologs per CEG.

e Percentage of detected CEGs that have more than one ortholog.

(b) BUSCO result

	E. crus-galli		*S.bicolor*		*S.italica*	
	Number	Percent	Number	Percent	Number	Percent
Complete(C)	1375	95.5%	1389	96.4%	1359	94.3%
Complete single-copy(S)	230	16.0%	1370	95.1%	1337	92.8%
Complete duplicated(D)	1145	79.5%	19	1.3%	22	1.5%
Fragmented(F)	25	1.7%	20	1.4%	42	2.9%
Missing(M)	40	2.8%	31	2.2%	39	2.8%

(c) Statistics of alignment rates of public ESTs and our assembled transcripts to the *E. crus-galli* genome assembly.

Query sequences	Median coverage (%)	Median identity (%)	Bases covered (%)	Percent of sequences with >90% base coverage (%)
74 public *E. crus-galli* ESTs	98.70	99.30	92.98	91.90
156,757 PASA assembled transcripts	99.93	99.99	99.86	99.84

Supplementary Table 4. Summary of transcriptome data by RNA-seq from *E. crus-galli* in this study.

(a) For *E. crus-galli* gene prediction

Tissue	ID	Read number	Total raw bases
whole plant	A2	29,778,572	2,977,857,200
whole plant	A6	30,264,710	3,026,471,000
whole plant	A8	29,128,768	2,912,876,800
whole plant	A10	31,004,534	3,100,453,400
whole plant	A12	33,892,022	3,389,202,200
whole plant	A14	28,955,916	2,895,591,600
whole plant	A16	30,155,088	3,015,508,800
whole plant	A18	28,555,232	2,855,523,200
whole plant	B2	21,624,910	2,162,491,000
whole plant	B4	21,443,844	2,144,384,400
whole plant	B6	19,477,752	1,947,775,200
whole plant	B8	19,110,314	1,911,031,400
whole plant	B10	19,918,316	1,991,831,600
whole plant	B12	23,320,984	2,332,098,400
whole plant	B14	21,589,774	2,158,977,400
whole plant	B16	21,006,202	2,100,620,200
whole plant	B18	22,125,290	2,212,529,000
Total		431,352,228	43,135,222,800

(b) Allelopathic experiments (*E. crus-galli* v.s. *O.sativa*)

**Tissue**	Experiments	Read number	Total raw bases
whole plant	M1	24,822,786	3,723,417,900
whole plant	M2	29,415,628	4,412,344,200
whole plant	M3	27,987,694	4,198,154,100
whole plant	C1	21,521,612	3,228,241,800
whole plant	C2	36,900,398	5,535,059,700
whole plant	C3	31,004,736	4,650,710,400

Supplementary Table 5. Numbers of non-coding RNAs in *E. crus-galli*.

Type		Number	Average Length
miRNA		785	138
rRNA		1890	628
sRNA		25	155
tRNA		2306	74
snRNA		463	155
snoRNA	CD-box	3185	104
	HACA-box	193	130
	total	3378	105

Supplementary Table 6. Percentage of repeat sequences in *E. crus-galli* and other grass genomes.

Super-family		***O. staiva***	***S. bicolor***	***Z. mays***	***B. distachyon***	***S. italica***	***E. crus-galli***
Retroelements	LTR	18.18	54.43	74.6	21.39	29.58	21.91
	LINEs	1.12	0.04	1	1.94	1.81	2.01
	SINEs	0.06	0	0	0	0.17	0.52
DNA transposons		12.96	7.46	8.6	4.77	9.38	7.96
Unknown (unclassified)		1.8	0.12	-	-	5.39	8.27
Total TEs		34.1	62	84.2	28.1	46.44	40.68

Supplementary Table 7. Primers used for qRT-PCR in this study.

Name of gene	Forward primer	Reverse primer
Tubulin	TGTTGTGAGGAAGGAAGCTG	GTGGCATTGTATGGCTCAAC
MAS	TGAACCTGGTGGGTCCATT	TCGACGTCGAGATGATGCAT
KSL4	TCATCCTCGTCTCACTTTCG	CCTTGCATTCCATGTTTGTC
