# Supplementary material for: Echinochloa crus-galli genome analysis provides insight into its adaptation and invasiveness as a weed
Source: Nat Commun. 2017 Oct 18;8:1031. doi: 10.1038/s41467-017-01067-5 (PMC5647321; doi:10.1038/s41467-017-01067-5)
Supplement: Supplementary file 2 — Description of Additional Supplementary Files [file 41467_2017_1067_MOESM2_ESM.pdf]

Description of Additional Supplementary Files

File Name: Supplementary Data 1
Description: Enriched pathway categories for DEGs of *E. crus-galli* during allelopathy against rice using the Mapman software.

File Name: Supplementary Data 2
Description: Expression patterns for genes in the three identified DIMBOA cluster copies in the *E. crus-galli* genome.

File Name: Supplementary Data 3
Description: The list of gene names in the phylogenetic trees showed in Supplementary Fig. 8.

File Name: Supplementary Data 4
Description: Statistics for each step during *E. crus-galli* genome assembly.
